# Human Infection with Avian Influenza A(H9N2) Virus, Cambodia, February 2021

**DOI:** 10.3201/eid2710.211039

**Published:** 2021-10

**Authors:** Samnang Um, Jurre Y. Siegers, Borann Sar, Savuth Chin, Sarika Patel, Seng Bunnary, Makara Hak, Sothy Sor, Oum Sokhen, Seng Heng, Darapheak Chau, Tum Sothyra, Asheena Khalakdina, Joshua A. Mott, Sonja J. Olsen, Filip Claes, Ly Sovann, Erik A. Karlsson

**Affiliations:** National Institute of Public Health, Ministry of Health, Phnom Penh, Cambodia (S. Um, S. Chin, D. Chau);; Institut Pasteur du Cambodge, Phnom Penh (J.Y. Siegers, E.A. Karlsson);; US Centers for Disease Control and Prevention, Phnom Penh (B. Sar);; World Health Organization Country Office, Phnom Penh (S. Patel, A. Khalakdina);; National Animal Health and Production Research Institute, Phnom Penh (S. Bunnary, T. Sothyra);; Food and Agriculture Organization of the United Nations Country Office, Phnom Penh (M. Hak);; Prevention and Control Disease Bureau, Provincial Health Department, Siem Reap, Cambodia (S. Sor); Provincial Office of Animal Health and Production, Siem Reap (O. Sokhen);; Cambodian Center for Disease Control, Ministry of Health, Phnom Penh (S. Heng, L. Sovann);; US Centers for Disease Control and Prevention, Bangkok, Thailand (J.A. Mott, S.J. Olsen);; Food and Agriculture Organization of the United Nations Regional Office for Asia and the Pacific, Bangkok (F. Claes)

**Keywords:** avian influenza, A(H9N2), respiratory infections, zoonoses, spillover, One Health, Cambodia, viruses, influenza

## Abstract

In February 2021, routine sentinel surveillance for influenza-like illness in Cambodia detected a human avian influenza A(H9N2) virus infection. Investigations identified no recent H9N2 virus infections in 43 close contacts. One chicken sample from the infected child’s house was positive for H9N2 virus and genetically similar to the human virus.

Low pathogenicity avian influenza virus subtype A(H9N2) is endemic in poultry in Asia, the Middle East, and Africa (*1*). These viruses do not cause mass mortality in poultry but can cause substantial negative economic impacts (*2*). H9N2 viruses also have zoonotic potential; 74 human infections were reported from 1998 through early 2021 (*1*,*3*,*4*), mainly in children with a history of poultry exposure. The internal gene cassettes of H9N2 viruses contribute to human adaptation of avian influenza viruses (AIV) strains, as exemplified by the fifth wave of the H7N9 epidemic in China (*5*). Because of broad host range, global distribution, and reassortment capability, risk assessments indicate existing H9N2 viruses as a moderate pandemic risk (*2*,*6*).

H9N2 viruses circulate endemically in poultry from Cambodia, as they do in Bangladesh, Vietnam, and China (*7*,*8*). Seventy-five percent of AIVs detected in chickens in Cambodia in live bird markets (LBMs) are H9N2 (*7*). Whereas serosurveys indicate persons in Cambodia with poultry contact are exposed to H9N2, active human infections have not been detected (*9*,*10*).

On February 26, 2021, a 3-year-old boy living in Prasat Bakong, near Siem Reap, Cambodia, was taken to an outpatient clinic for influenza-like illness with onset of symptoms on February 24 (Figure). A nasopharyngeal sample obtained as part of influenza-like illness sentinel surveillance tested positive for influenza A at the National Institute of Public Health (Phnom Penh, Cambodia) but was unsubtypable for human seasonal, H5, or H7 subtypes (Appendix 1 reference *11*). The sample tested positive for H9N2 at the National Influenza Centre, Institute Pasteur du Cambodge (Phnom Penh) (*7*); a second specimen obtained on March 3 confirmed H9N2 infection. Viruses in the 2 samples were successfully isolated in embryonated chicken eggs and confirmed as H9N2; the second isolate was named A/Cambodia/21020301/2021. Hemagglutination inhibition testing confirmed infection by seroconversion. The serum sample taken on March 3 (7 days postonset) tested negative and then tested positive on March 10 (14 days postonset; hemagglutination inhibition = 240) (Appendix 1 reference *12*).

**Figure:**
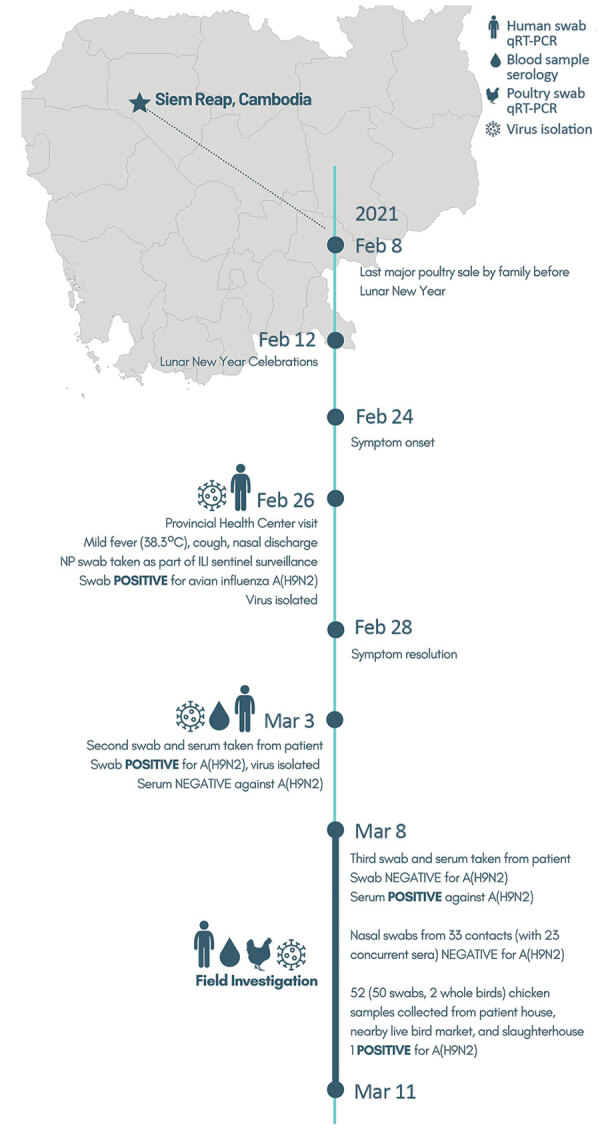
Locations of human infection and timeline of key events in the One Health investigation, Cambodia, February–March 2021. Key points and findings are indicated next to dates. Sampling and testing results are indicated by icons (in key). ILI, influenza-like illness; NP, nasopharyngeal; qRT-PCR, quantitative reverse transcription PCR.

A joint One Health investigation was undertaken during March 8–11, 2021, by the Cambodia Centers for Disease Control and Ministry of Health, National Animal Health and Production Research Institute, and provincial divisions with support from the United States Centers for Disease Control and Prevention, the World Health Organization, Food and Agriculture Organization, and Institute Pasteur du Cambodge. The infected child lives in a mainly agrarian village ≈20 km from Siem Reap encompassing 121 households and 502 inhabitants; 80% of families conduct small-scale backyard poultry farming. The child’s residence includes a chicken enclosure containing ≈50 chickens surrounded by nylon net. The child played within the enclosure and accompanied adults during feeding time. Poultry production, trading, and AIV prevalence in LBMs increase during national festival periods (*7*); a major sale took place on February 8, 2021, before Lunar New Year. 

Forty-three close contacts were identified in 9 households (21 persons <15 and 22 persons ≥15 years of age; 53% female). Nine persons from 5 households reported respiratory symptoms immediately before or during the investigation. Nasal swab samples from 33/43 contacts (including all symptomatic persons), and concurrent serum samples from 23/43 contacts (including 8/9 symptomatic persons) showed neither H9N2 infection nor seroconversion. Health education on safe poultry farming practices was provided to representatives of close contact families.

Investigations of poultry in the index house, village, local LBMs, and slaughterhouses yielded 50 tracheal/cloacal samples and 2 whole birds. One H9N2 virus was detected from a chicken at the infected child’s house, isolated in embryonated chicken eggs, and subsequently named A/chicken/Cambodia/f0318251/2021. No poultry die-offs were reported in February 2021.

We sequenced the hemagglutinin and neuraminidase genes with Oxford Nanopore Technologies (Nanopore, https://nanoporetech.com) (Appendix 1 Figure). The hemagglutinin genes of A/Cambodia/21020301/2021 and A/chicken/Cambodia/f0318251/2021 (GISAID, https://www.gisaid.org; accession nos. EPI4858549, EPI1858551) clustered with G9/BJ94 lineage viruses from southern China from 2018. Neuraminidase genes (accession nos. EPI4858550, EPI1858552) clustered with G9/BJ94 lineage viruses from Laos from 2019. Overall, genetic distances between human and chicken viruses support a possible recent shared ancestor consistent with household chickens as the source of exposure.

Seroprevalence studies in rural Cambodia populations show neutralizing antibodies against H9N2 viruses of 1.1%–2.6%, indicative of undetected infections (*9**,**10*: Appendix 1 reference *13*), similar to avian-exposed persons elsewhere (Appendix 1 reference *14*). Therefore, the true burden of human H9N2 virus infections is likely higher than observed. Several human infections with H9N2 were reported in 2020, coinciding with the coronavirus pandemic (Appendix 1 reference *15*), when global human seasonal influenza declined, suggesting that sentinel surveillance systems continued to detect seasonal and zoonotic viruses (Appendix 1 reference *16*).

Endemic H9N2 and other AIVs in poultry remain a concern for zoonotic infection. An interdisciplinary One Health approach is warranted to curb continuing expansion and emergence. Early detection and control in animal populations, enhanced biosafety, candidate vaccines, and prompt antiviral treatment might mitigate the risks of reassortment and continued evolution that could result in H9N2 viruses with increased mammalian adaptation or human-to-human transmission potential.

Appendix 1 Additional information on human infection with avian influenza A(H9N2) virus, Cambodia.

Appendix 2 GISAID sequences used for investigation of human infection with avian influenza A(H9N2) virus, Cambodia.
