# Supplementary material for: Human Infection with Avian Influenza A(H9N2) Virus, Cambodia, February 2021
Source: Emerg Infect Dis. 2021 Oct;27(10):2742–5. doi: 10.3201/eid2710.211039 (PMC8462329; doi:10.3201/eid2710.211039)
Supplement: Appendix 1 — Additional information on human infection with avian influenza A(H9N2) virus, Cambodia. [file 21-1039-Techapp-s1.pdf]

Article DOI: https://doi.org/10.3201/eid2710.211039

Human Infection with Avian Influenza A(H9N2) Virus, Cambodia, February 2021

Appendix 1

Additional References

11. Horwood PF, Karlsson EA, Horm SV, Ly S, Heng S, Chin S, et al. Circulation and characterization of seasonal influenza viruses in Cambodia, 2012–2015. Influenza Other Respir Viruses. 2019;13:465–76. PubMed https://doi.org/10.1111/irv.12647

12. World Health Organization. Manual for the laboratory diagnosis and virological surveillance of influenza; 2011.

13. Gray GC, Krueger WS, Chum C, Putnam SD, Wierzba TF, Heil GL, et al. Little evidence of subclinical avian influenza virus infections among rural villagers in Cambodia. PLoS One. 2014;9:e97097. PubMed https://doi.org/10.1371/journal.pone.0097097

14. Khan SU, Anderson BD, Heil GL, Liang S, Gray GC. A systematic review and meta-analysis of the seroprevalence of influenza A(H9N2) infection among humans. J Infect Dis. 2015;212:562–9. PubMed https://doi.org/10.1093/infdis/jiv109

15. World Health Organization. Influenza monthly risk assessment summary. 2021 [cited 2021 April 16]; https://www.who.int/influenza/human_animal_interface/HAI_Risk_Assessment/en/

16. Sovann LY, Sar B, Kab V, Yann S, Kinzer M, Raftery P, et al. An influenza A (H3N2) virus outbreak in the Kingdom of Cambodia during the COVID-19 pandemic of 2020. Int J Infect Dis. 2021;103:352–7. PubMed https://doi.org/10.1016/j.ijid.2020.11.178

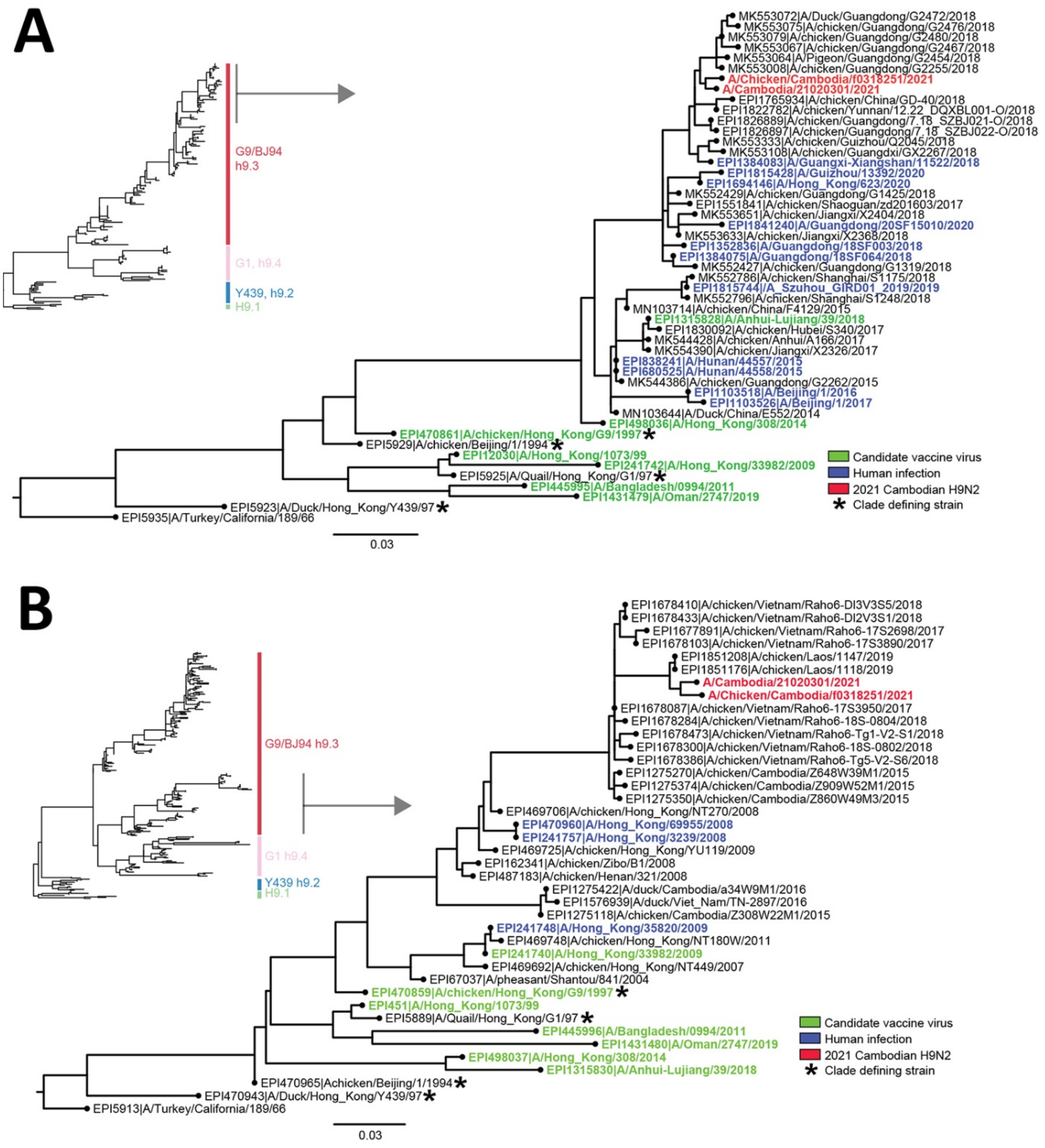

Figure. Maximum likelihood phylogenetic tree of hemagglutinin (A) and neuraminidase (B) genes of avian influenza A(H9N2) viruses detected in human and chicken specimens, Cambodia, 2021. Sequences were aligned using MAFFT v7.450 in Geneious Prime 2021.0.1 against 47 and 38 A/H9N2 HA and NA sequences, respectively, downloaded from the GISAID and Genbank database (Appendix 2, https://wwwnc.cdc.gov/EID/article 27/10/21-1039-App2.xlxs). Maximum Likelihood (ML) phylogenetic trees were inferred with IQ-Tree (v2.0.3) using the best-fit nucleotide substitution model (FLU+I+G4) and visualized using FigTree (v1.4.4) and ggree (v2.4.1). Phylogenetic support was estimated using 1,000 ultrafast bootstrap replicates. Candidate vaccine viruses are indicated in bold green, human A(H9N2)

viruses in bold blue, and 2021 Cambodian H9N2 viruses in bold red. Clade defining strains are indicated with an asterisk. Scale bars indicate the number of nucleotide substitutions per site. Genbank accession and GISAID isolate ID numbers are provided in Appendix 2.
